# Perioral Clonal Variant of Seborrheic Keratosis: Diagnostic Challenges

**DOI:** 10.7759/cureus.36381

**Published:** 2023-03-20

**Authors:** Nitya K, Karthikeyan M, Ramya R, Anusha JA, Vikram S A

**Affiliations:** 1 Oral and Maxillofacial Pathology, Saveetha Dental College & Hospital, Chennai, IND; 2 Oral and Maxillofacial Surgery, Sathya Sai Medical College & Research Institute, Chennai, IND; 3 Department of Oral Biology, Saveetha Dental College & Hospital, Chennai, IND; 4 Oral Medicine and Radiology, Sri Mookambigai Institute of Dental Sciences, Chennai, IND; 5 Oral Pathology and Microbiology, College Of Dental Sciences, Davangere, IND

**Keywords:** horn cyst, ki- 67, pan ck, clonal sk, seborrheic keratosis, gene expression, uv radiation

## Abstract

Seborrheic keratosis is the most common benign tumour of epithelial origin, whose incidence increases with the advance of age. The aetiology of SK is not completely known but exposure to UV radiation may be an associated factor. Most of the cases have a rough, verrucous appearance, slightly elevated, and black or brown in colour. They are usually painless but at times present with pus discharge or ulceration.

## Introduction

Seborrheic keratosis (SK) is a very common non-cancerous lesion, mostly appearing on sun-exposed areas of skin especially the face, trunk, back and chest. It occurs mainly by hyperproliferation of immature keratinocytes due to factors like sun exposure or genetic aetiology [1]. They appear frequently in the elderly population without any gender predilection. They appear as exophytic lesions with a verrucous/rough appearance. While most of the lesions are usually asymptomatic, at times they can exhibit features of malignant transformation such as pain, rapid growth, itching, bleeding, etc [1,2]. Here we discuss a case of a long-standing asymptomatic lesion, which was diagnosed as seborrheic keratosis (clonal variant).

## Case presentation

A 65-year-old female patient reported a complaint of a black-coloured growth present on her right cheek region for the past three years. Initially, it was a small pigmented growth, which has slowly increased in size. The patient had a previous history of similar growth (but it was slightly smaller in size) on which liquid nitrogen was applied in a spray. The growth was reduced after a week and within one month, the black mass has completely fallen off. No other significant medical history was elucidated by the patient.

**Figure 1:**
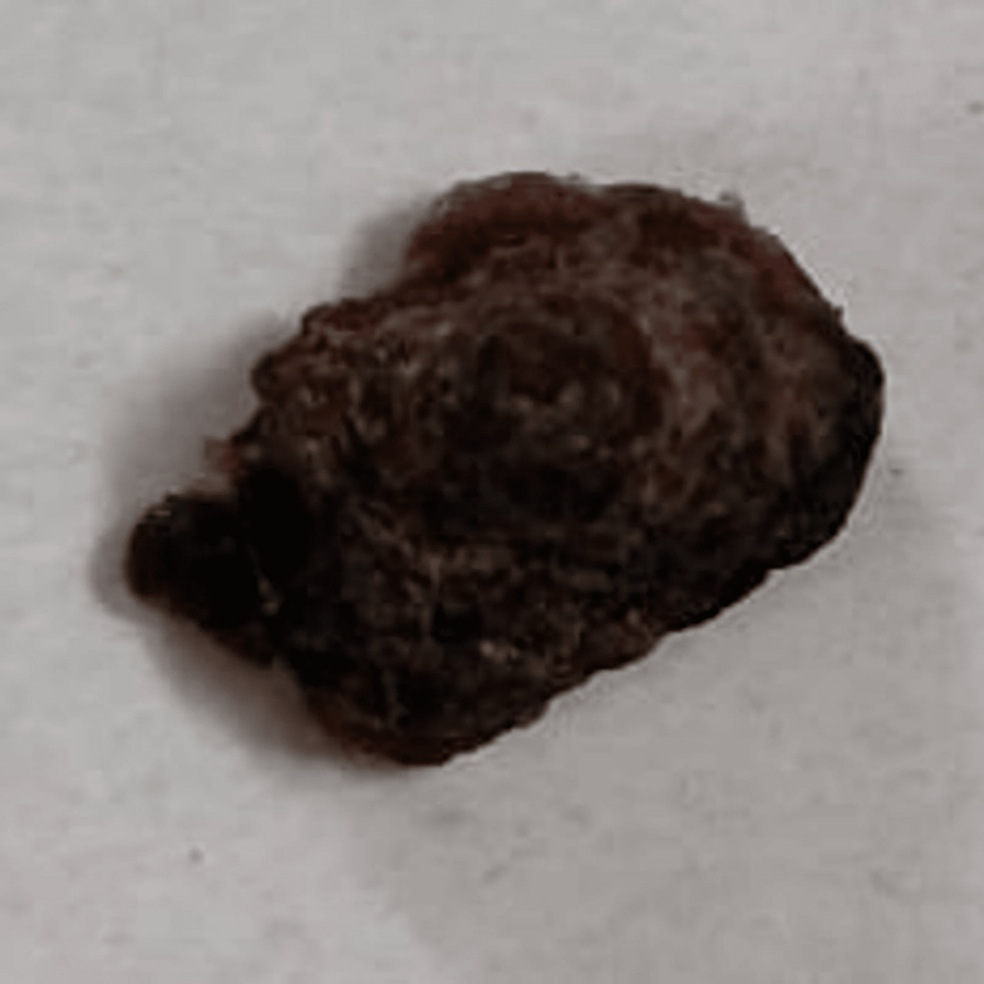
Surgically excised lesion with shave biopsy; it appears blackish and rough with a verrucous surface.

On inspection, a well-defined, solitary growth of 1.5 X 1 mm was seen on the right cheek region. The colour appeared to be blackish with a rough and verrucous surface. No bleeding or pus discharge was noted. The skin adjacent to the growth appeared to be normal. On palpation, all the findings from the inspection were confirmed. The growth was firm in consistency, non-tender, and non-fluctuant with no evidence of bleeding and pus discharge (Figure 1). So, a provisional diagnosis of seborrheic keratosis was made, with a differential diagnosis of squamous cell carcinoma and basal cell carcinoma.

**Figure 2:**
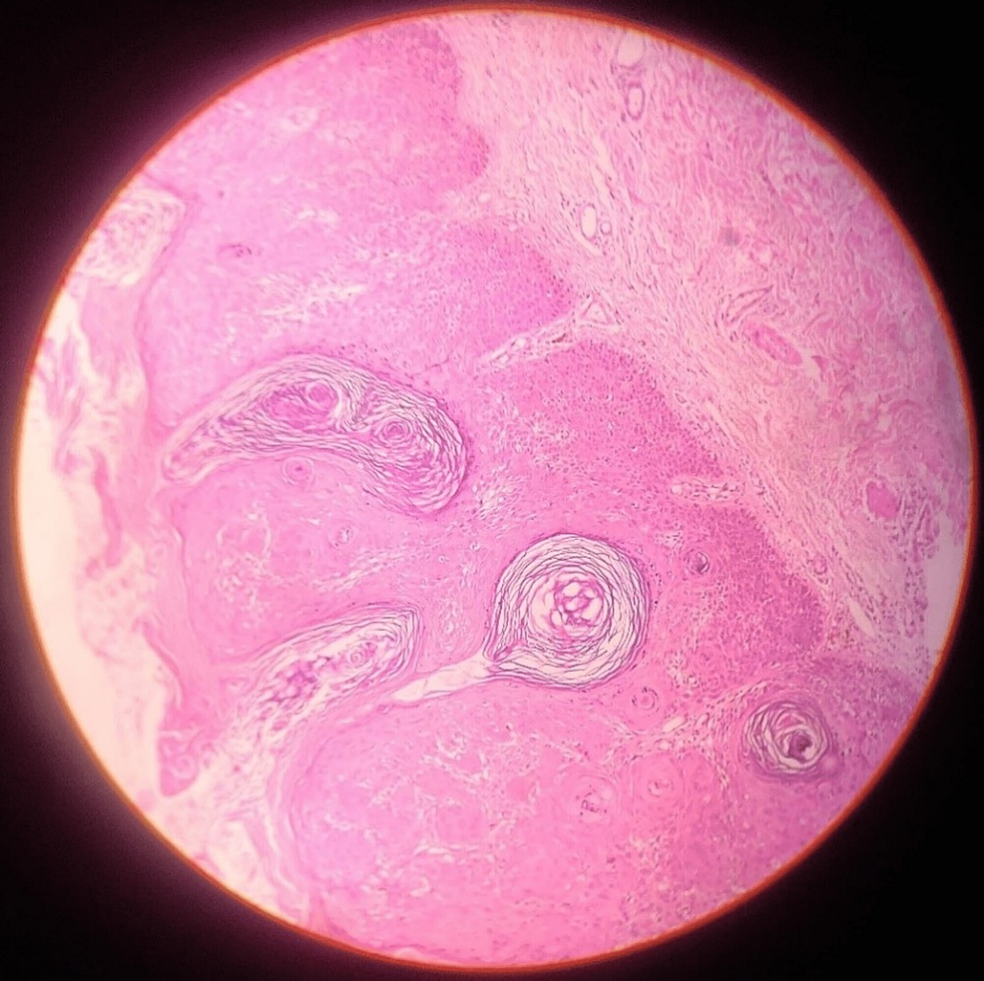
Hematoxylin and eosin-stained tissue section showing hyperkeratosis, squamous eddies, horn cysts

To rule out the malignant transformation, a shave biopsy was conducted and the specimen was submitted for histopathological analysis. The hematoxylin and eosin-stained tissue section revealed an intraepidermal lesion with a flat base showing squamous eddies and basaloid proliferation of keratinocytes. Hyperkeratosis and the presence of pseudo-horn cysts were also noted (Figure 2). No features of atypia or malignancy were seen. Topical application of tazarotene and antibiotics were prescribed for one week and the patient was followed up after six months for review; to date, no evidence of growth was noted.

**Figure 3:**
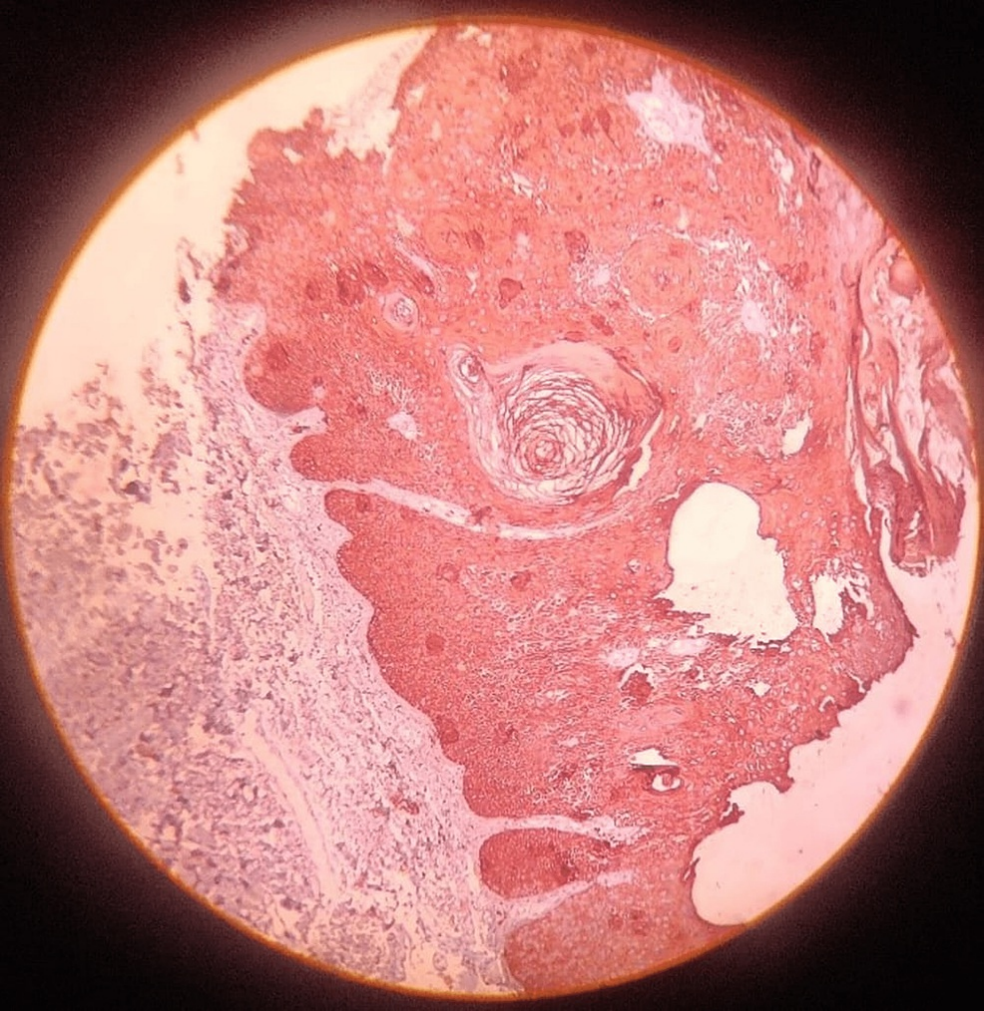
Immunohistochemistry staining with pan-cytokeratin

**Figure 4:**
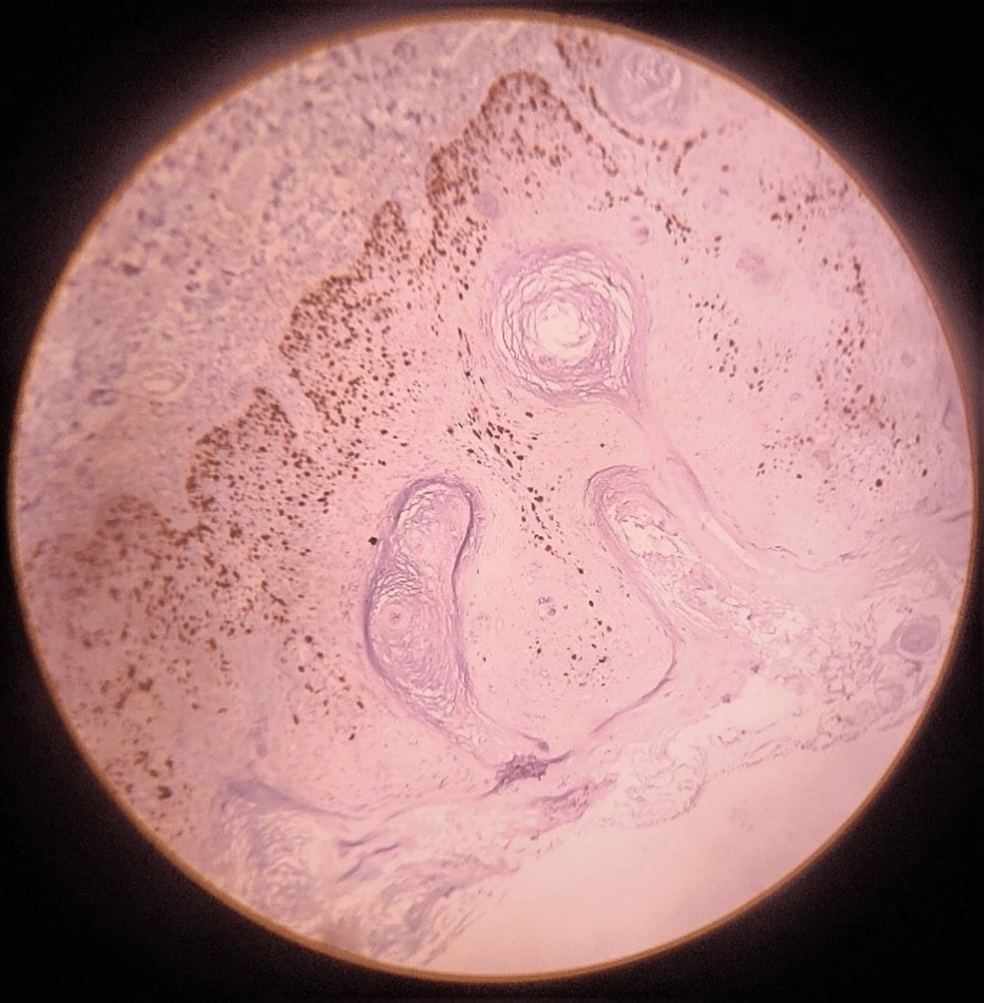
Immunohistochemistry staining of Ki-67 expression

As large-sized seborrheic keratosis in elderly patients can undergo a malignant transformation which is reported in the literature, immunohistochemistry (IHC) using pan-cytokeratin and Ki-67 was performed for confirmation (Figures 3-4). 

## Discussion

Seborrheic keratosis (SK) is one of the most common epidermal tumours that occur in adults. The common site of occurrence of SKs are the face, trunk, and neck; trunk they follow the Langer's line. They are mostly uncommon in palms and soles.

The exact pathogenesis of seborrheic keratosis is unclear. They are referred to as a sign of ageing skin which is due to long-standing ultraviolet (UV) exposure [3]. Though SK is thought to be genetically stable, somatic alterations like FGFR3, PIK3CA, HRAS, KRAS and EGFR mutations prevail in SK that are responsible for malignant transformations [4,5]. A hypothesis postulating human papillomavirus aetiology in SK involving the genital region has also been reported in the literature. The gene amyloid precursor protein (APP) expression was found to be expressed higher in the case of UV-exposed SK sites and in increased age groups [6,7]. SK also have acquired oncogenic mutations in tyrosine kinase/AKT-signaling cascades associated with hypersensitivity to AKT inhibition [8]. Results of recent studies suggest that seborrheic keratosis has a tendency for malignant transformation around 1.4 to 7%. The results of previous studies also state that basal cell carcinoma (BCC) and squamous cell carcinoma (SCC) tend to occur as a complication of SK or in longstanding cases [9]. Other tumours like adenocarcinoma, trichilemmal carcinoma, eccrine poro-carcinoma, primary cutaneous ganglioneuroma were also reported in the literature but BCC was the most common neoplasm followed by SCC [10].

Common dermoscopic features of SK are fissures and ridges, hairpin vessels with halo, comedo-like openings and milia-like cysts. The histopathologic findings include a papillomatous or verrucous-like epidermis, dermal capillaries being enlarged, pseudo horn cysts and intraepidermal cysts [11]. Seborrheic keratosis shows varied clinical and histopathological subtypes such as hyperkeratotic, acanthotic, reticular type, clonal variant, irritated SK, melanoacanthoma and features resembling verrucous SK with keratoacanthoma [12].

Dermatosis papulosa nigra is a clinical variant of SK with numerous tiny lesions and is most commonly seen in patients with Fitzpatrick skin type III to VI. The appearance of multiple eruptive seborrheic keratoses is referred to as the Leser-Trelat sign (inflammation of existing SK during the treatment of malignancies) and is considered a feature of paraneoplastic syndrome. Tumours such as lung cancer, oesophagal carcinoma, nasopharyngeal carcinoma, mycosis fungoides, Sézary syndrome, and plasmacytoma are found to be associated with paraneoplastic disorder [13,14].

The hyperkeratotic type is the most common subtype, which is characterized by increased acanthosis with mild hyperkeratosis and papillomatosis. Horny projections are seen and are referred to as pseudo-horn cysts, whereas true horn cysts are made of complete keratinization with granular layers surrounding them. Reticular or adenoid variants are characterized by hyperkeratosis and papillomatosis with reticular acanthosis. Most of them are pigmented and are common in sun-exposed skin areas [15]. In the clonal variant, acanthosis and papillomatosis with hyper orthokeratosis are seen. The tumour cells appear spindle-shaped, and islands of basaloid cells are found. The differential diagnosis to clonal SK is pagetoid Bowen disease, so in such cases, immunohistochemistry is helpful in distinguishing between the two. Cytokeratin-10 is highly expressed in the clonal variant whereas increased Ki-67-positive cells are indicators of pagetoid Bowen disease. In the present case, pan-cytokeratin expression was found to be intensely stained and Ki-67 was found to be minimally positive. In irritated seborrheic keratosis, the eosinophilic tumour cells appear spindle-shaped in a whorl-like fashion. It is difficult to distinguish irritated SK from cutaneous squamous cell carcinoma. So immunohistochemistry using U3 IMP3 and Bcl-2 will help in understanding the nature of the lesion and differentiating them [16,17].

Features of this condition include the proliferation of basaloid cells with or without hyperkeratosis, the presence of numerous melanocytes, as well as melanophages in the dermis in melanoacanthoma and these are very rarely seen on the oral mucosa. In the case of verrucous SK with keratoacanthoma-like features, verrucous proliferation with keratoacanthoma histologic features is seen. They show features like craters with keratin plugging, well-differentiated epidermis etc. The role of HPV is noted in these cases [15,16].

Immunosuppression is also one of the factors for developing secondary tumours in SK. This is mainly due to an impaired immune system leading to the proliferation of CD4 cells and mutant cells. Also in elderly patients, B & T cells lose their ability to proliferate as ageing progresses leading to loss of lymphocyte population. Tumour transformation is more likely to be present in SK-exposed areas, thus leading to the probability that sun/UV exposure is influential for the development of secondary tumours in Seborrheic keratosis [17].

Clinical features like ulceration and erythema around SK lesions have a possible role in tumour formation. Criteria for diagnosis of tumour in SK are cytologic atypia, mitosis with similar features of SK, hyperkeratosis, parakeratosis, papillomatosis and pseudo horn cyst [18,19].

In secondary tumours with SK, features such as ulceration, cytologic atypia, mitosis, solar elastosis and malignant horn (eosinophilic stratum corneum with hyperchromatic brick-like parakeratosis) are seen. Other histological features include acantholysis, dyskeratosis, spongiosis, squamous eddies, necrosis and basal cell vacuolization. Clinicians and pathologists should always be aware of the possibility of squamous cell carcinoma or basal cell carcinoma arising in SK and should have an increased suspicion for it [19].

In a clonal variant of seborrheic keratosis, pan-cytokeratin is used for supra basaloid differentiation stages and Ki-67 is used as a cellular marker for proliferation. They play a vital role in differential diagnosis, treatment planning and prognosis of the disease. Though this lesion was removed for cosmetic reasons, long-term observation is necessary. Various treatment options include cryosurgery, curettage, surgical dissection, laser ablation and electrodesiccation; also, local or systemic drug delivery using tazarotene, calcipotriene, and ammonium lactate can be used. Scarring and recurrence of seborrheic keratosis are the major disadvantages of the treatment.

As SK has a close association with malignant lesions such as squamous cell carcinoma and malignant melanoma, obtaining a thorough history, proper evaluation and close follow-up are necessary in these cases.

## Conclusions

Seborrheic keratosis as such does not require any treatment unless for cosmetic reasons, but in cases of early detection and appropriate diagnosis, these cases would have an influence on the prognosis and treatment planning. So both clinical and histopathological investigations should always be carried out and given importance with frequent follow-up of the patient to prevent any malignant transformations.
